# Functional sterol improves breast milk quality by modulating the gut microbiota: A proposed opinion for breastfeeding mothers

**DOI:** 10.3389/fnut.2022.1018153

**Published:** 2022-11-08

**Authors:** Jeanette Irene Christiene Manoppo, Fahrul Nurkolis, William Ben Gunawan, Gilbert Ansell Limen, Ronald Rompies, Joko Purnomo Heroanto, Hans Natanael, Sardito Phan, Krisanto Tanjaya

**Affiliations:** ^1^Department of Pediatrics, Faculty of Medicine, Sam Ratulangi University, Manado, Indonesia; ^2^Department of Pediatrics, Prof. R. D. Kandou General Hospital, Manado, Indonesia; ^3^Biological Sciences, Faculty of Sciences and Technology, State Islamic University of Sunan Kalijaga (UIN Sunan Kalijaga Yogyakarta), Yogyakarta, Indonesia; ^4^Department of Nutrition Science, Faculty of Medicine, Diponegoro University, Semarang, Indonesia; ^5^Medical Programme, Faculty of Medicine, Sam Ratulangi University, Manado, Indonesia; ^6^Medical Programme, Faculty of Medicine, Universitas Brawijaya, Malang, Indonesia

**Keywords:** functional sterol, breast milk, phytosterols, gut microbiota, breastfeeding, functional food

## Introduction

Breast milk is the best food for an infant in their early life. This is not only because breast milk has complete nutrients that the baby needs but also contains immunological substances that can protect babies from various kinds of infections. Exclusive breastfeeding for the first 6 months as recommended by the WHO can prevent various conditions, such as malnutrition and allergies, improve the cognitive development of the baby, and reduce infant mortality (1–3). Feeding breast milk can create a bond between mothers and their babies (4).

The overall quality of breastmilk is highly conserved, but it is directly influenced by maternal dietary habits (5). Unlike formulas, the composition of breast milk will change based on various factors. The content of breast milk can be influenced by genetics, environment, food intake of the mother, and nutritional and infective status (6). Food intake containing macronutrients turns into liquid breast milk when the food is digested in the body and then carried by blood cells throughout the body. The intake of macronutrients in food during breastfeeding needs to be increased because the mother needs extra energy for recovery after childbirth and the metabolic process of milk formation. The food consumed by the mother indirectly affects the quality and the amount of milk produced (7).

The gut microbiota is formed in the early days of life and could directly affect the development of the immune system later in life (8). The gut microbiota of the mother is one of the essential factors that influence the microbiota of an infant through active transport from the mother's gastrointestinal (GI) tract to the human milk using the intramammary pathway. Therefore, human milk produced by healthy women contains several microbiotas, including *Streptococcus* and *Staphylococcus*, which were successfully identified as the dominant genera (9). The function of gut microbiota is highly dependent on the dietary compounds found in the GI tract. One of the factors influencing gut microbiota is a sterol, and due to its functional ability, it is called a functional sterol. Plant sterols, also known as phytosterols or functional sterols, play an important role in the modulation of gut microbiota (10) and are therefore considered potential functional sterols based on the abovementioned health effects. The main purpose of this opinion article is to interpret the latest findings about potential sterols as functional foods in breastfeeding mothers in improving the quality of breast milk by modulating the gut microbiota.

## Functional sterol

Functional foods are foods that possess constructive effects on target functions in humans, beyond nutritional effects, with aim of promoting health and well-being and/or reducing the risk of chronic disease (11). Phytochemicals are bioactive compounds generated from secondary plant metabolism in response to environmental changes, including carotenoids, indoles, glucosinolates, organosulfur compounds, phytosterols, polyphenols, and saponins (12). Plant sterols and stanols, which are collectively referred to as plant sterols or phytosterols of the phytochemicals, are cholesterol homologs that are found naturally in all plant foods including nuts, fruits, vegetables, grains, legumes, and seeds (13). The highest concentration of phytosterols is found in unrefined plant oils, including vegetables, nuts, and olive oils, such as sesame, safflower, soybeans, peas, macadamia, and almonds (14).

As a plant-derived fatty compound, plant sterols are non-nutritive compounds and represent the greatest portion of unsaponifiable matter in plant lipids (15). The characteristic of their steroid skeleton is a saturated bond between C-5 and C-6 of the sterol moiety and the attachment of an aliphatic side chain to the C-17 atom, along with a hydroxyl group attached to the C-3 atom. The only difference in its structure from cholesterol is methyl or an ethyl group at C-24. The saturation of these plant sterols generates plant stanols that have no double bond in the sterol ring (14, 16).

The most frequently found plant sterols in food are sitosterol and campesterol which constitute about 60 and 35%, respectively. The normal western-type diet contains about 200–500 mg of cholesterol, 200–400 mg of plant sterols, and about 50 mg of plant stanols (16). The intestinal absorption rate of plant sterols is estimated to range from 0.4 to 5% and for their saturated counterparts, it is estimated to range from 0.02 to 0.3% (17). Consequently, the serum concentration of plant sterol is about 10 times higher than plant stanol. In addition, due to their lower absorbability rate, and the fact that they are not synthesized in the human body results in 1,000 times lower concentrations than cholesterol (18–20).

Phytosterols and cholesterol are taken up in micelles to be absorbed and then interact with the intestinal brush layer, enabling absorption into enterocytes. The absorption of cholesterol is competitively inhibited by phytosterols (21). There is increasing scientific evidence showing the health benefits of plant sterols, such as reducing total low-density lipoprotein (LDL) cholesterol levels (22) and possessing antioxidant, antiulcer, immunomodulatory, antibacterial, antifungal, and antiplatelet effects (23, 24). Yasin showed that the consumption of Moringa leaves rich in phytosterols can increase breast milk production in breastfeeding mothers (25).

## The modulation of gut microbiota improves breast milk quality

Maternal microbiota influences the early-life microbiota and has a lasting impact on childhood development, which can regulate the health of a person (26). Kalbermatter et al. and Nyangahu and Jaspan highlighted that the innate immune system of a growing fetus can be effectively influenced by the mother's microbiota during pregnancy (27, 28). We proposed that maternal gut microbiota may affect the quality of breast milk. Certain maternal gut microbiota compositions may promote the secretion of immunoglobulin A (IgA) in breast milk (29). Secretory IgA is the main antibody found in breast milk and plays an important role in an infant's growth (30). Moreover, Avershina et al. suggested that there was a “microbiota sharing” across the maternal gut and breast milk although in low quantity (31). The diet type of mothers, especially one that is rich in fibers or proteins, can modify the microbiota profile and diversity of breast milk (32). In addition, *Staphylococcus*, lactic acid bacteria, and *Streptococcus* are the most abundant bacteria in colostrum (33). Therefore, regulating maternal gut microbiota as the precursor of breast milk microbiome may present chances to eventually improve the health and development of the infant's microbiome which ultimately improves the quality of breast milk. Based on this opinion, in the future, further clinical evidence is needed to explore gut microbiota with breast milk quality.

## Functional sterol in modulating gut microbiota

Several studies highlighted that sterol may modulate gut microbiota, resulting in beneficial health effects. Phytosterols from sweet potatoes regulated gut microbiota and prevented breast cancer by upregulating the abundance of *Bacteroides*, decreasing *Firmicutes*, and increasing the production of short-chain fatty acids (SCFAs) (34). These changes are beneficial since microbial composition will also change during lactation as evidenced by an increase in *Proteobacteria* and a decrease in *Firmicutes* (35). Stigmasterol, which is one of the most abundant plant sterols, showed anti-inflammatory effects, restored gut microbiota equilibrium, and enhanced the production of SCFAs mainly butyrate (36). Studies using *in vitro* model found that plant sterol changed the microbiota composition in the colon by increasing *Parabacteroides, Synergistaceae*, and *Lachnospiraceae*, along with a higher sterol metabolism and organic acid byproducts (10). Cuevas-Tena et al. (37) observed an increase in the *Erisipelotrichaceae* and *Eubacterium*. B-sitosterol is the most abundant plant sterol found in breast milk. B-sitosterol improves the expression of the JAK2/STAT5 signaling pathway by activating the GH axis and inhibiting SOCS2, further promoting the synthesis of β-casein. It promotes mTOR expression by activating IGF-1 and HIF-1α/EPO and increasing the downstream factor of mTOR S6K1, thereby influencing the synthesis of β-casein and aiding in infant growth (38). In addition, β-sitosterol increases the diversity of *Staphylococcus* and *Streptococcus*, which are found in colostrum and are very nutritious for babies (33). Campesterol showed a beneficial role in preventing metabolic syndrome and inflammation (39). Moreover, the supplementation of plant sterols may also increase the abundance of *Firmicutes* and the production of SCFAs in obese subjects based on an *in vitro* model (40), which may be useful for obese breastfeeding mothers. An *in vivo* study observed that phytosterol esters increased the abundance of *Bacteroidetes* and *Anaerostipes* while preventing non-alcoholic fatty liver disease (41), attenuating inflammation, and modulating gut microbiota (42). The consumption of phytosterol esters may also increase both monounsaturated and polyunsaturated fatty acid levels (43). The transformation of unabsorbed plant sterols, which is similar to that of cholesterols, plays a significant role in many biological processes related to health due to the modulation of gut microbiota and the produced metabolites (44).

## Future prospects by optimizing the consumption of functional sterol

Dietary habits are strongly associated with the composition and function of gut microbiota and will directly affect the health of humans (37). Natural plant sterol is easy to consume daily, such as in vegetables, nuts, cereal, seeds, some fruits, and legumes. Therefore, functional food contains functional sterol which has a cholesterol-lowering ability and could be consumed together with daily food. Based on the study conducted by Martianto et al. functional sterol, eaten together with daily food lowered the risk of cholesterol-related diseases (45) and improved the modulation of gut microbiota and the produced metabolites (44). Unlike dietary cholesterol, sterol's intestinal absorption rate is only 2–3%, while dietary cholesterol's intestinal absorption rate is 20–80%. Moreover, the cholesterol profile levels of mothers were associated with breast milk cholesterol concentrations, reflecting its important implications for the growth and health of the offspring (46). Cuevas-Tena et al. (44) showed that functional sterol is more preferred as the substrate for gut microbiota. Functional sterols, which exhibited prebiotic properties, may also become an alternative to probiotics in increasing breast milk quality since both of them have gut modulation activity (47, 48). Functional sterols include β-sitosterol, stigmasterol, sitosterol, campesterol, and phytosterol esters and are abundant in rice bran, wheat seeds, peanuts, corn oil, and soybeans (38, 49). Functional food researchers may also be interested in developing ready-to-eat products from abundant ingredients and functional sterols such as cookies through isolation and the application of phytosterol delivery nanotechnology, which may result in the higher bioavailability of functional sterols.

## Conclusion

**Figure 1:**
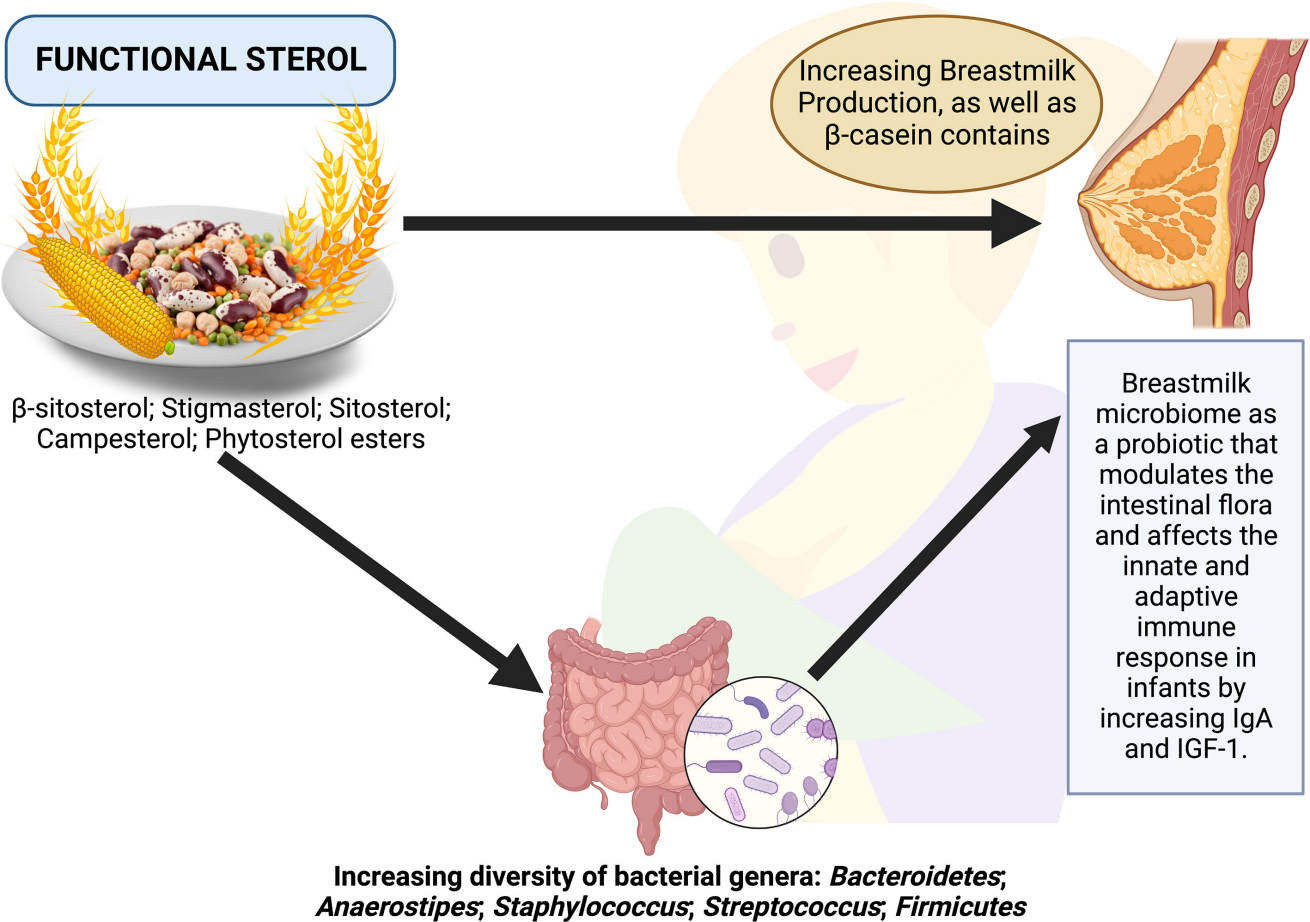
Possible scheme to improve breast milk quality through functional sterols via the modulation of the gut microbiota.

Functional sterols are plant-based sterols or phytosterols, including β-sitosterol, stigmasterol, sitosterol, campesterol, and phytosterol esters, which have the potential to be good agents in improving the quality of breast milk, directly or indirectly by modulating the composition of gut microbiota (Figure 1). Consumption of functional sterols increases the diversity of bacteria, such as *Bacteroidetes, Anaerostipes, Staphylococcus, Streptococcus*, and *Firmicutes*, which results in an improvement in the quality of colostrum and microbiome diversity in the breast milk of nursing mothers. The breastmilk microbiome is a probiotic environment that modulates the intestinal flora and affects the innate and adaptive immune response in infants (Figure 1). Breastfeeding mothers should consume functional sterols to improve the quality of breast milk given to their infants to aid in their growth. Some food sources of functional sterols include wheat seeds, peanuts or legumes, corn, and soybeans (Figure 1). Furthermore, in the future, there is a need for functional food specifically designed to enrich functional sterols through various processing methods and innovations. On the other hand, clinical research examining the effects of foods rich in functional sterols in modulating maternal gut microbiota and their relationship with breast milk quality is important.

## Author contributions

JM, FN, WG, GL, JH, HN, SP, and RR contributed to the conception and design of opinion studies and drafting manuscripts in advance. JM, FN, WG, and KT edited, revised, and approved the final version of the submitted manuscript. All authors contributed to the article and approved the submitted version.

## Conflict of interest

The authors declare that the research was conducted in the absence of any commercial or financial relationships that could be construed as a potential conflict of interest.

## Publisher's note

All claims expressed in this article are solely those of the authors and do not necessarily represent those of their affiliated organizations, or those of the publisher, the editors and the reviewers. Any product that may be evaluated in this article, or claim that may be made by its manufacturer, is not guaranteed or endorsed by the publisher.
